# An unusual presentation of cirrhotic pleural effusion in a patient with no ascites: a case report

**DOI:** 10.4076/1757-1626-2-6767

**Published:** 2009-08-10

**Authors:** Savio John, Manju P Paul, Uma K Murthy

**Affiliations:** Department of Medicine, SUNY Upstate Medical University750 E Adams Street, Syracuse, NY, 13210USA

## Abstract

Pleural effusion that develops in a patient with cirrhosis and portal hypertension, in the absence of cardiopulmonary disease, is termed hepatic hydrothorax. Hepatic hydrothorax very rarely presents in the absence of ascites. Although the exact mechanism is somewhat controversial, pleural effusion occurs when ascitic fluid moves through diaphragmatic defects which are opened up by increased intra-abdominal pressure. We report a case report of cirrhotic pleural effusion in a patient with no clinical or radiographic evidence of ascites and discuss the pathogenesis, diagnosis and management of this condition.

## Case presentation

Pleural effusion of usually greater than 500 ml that develops in a patient with cirrhosis and portal hypertension, in the absence of cardiopulmonary disease, is termed hepatic hydrothorax.

**Figure 1.**
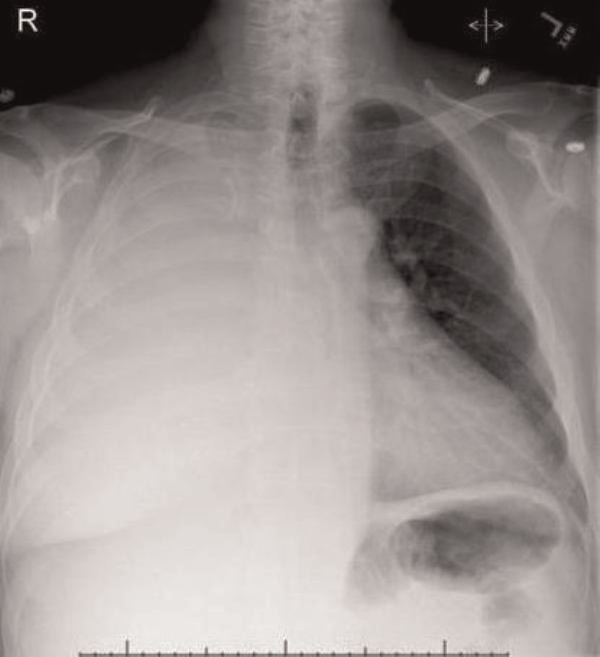
Chest X-ray showing right sided pleural effusion.

We report the case of a 56-year-old Caucasian male with hypertension and progressive painless jaundice who presented with shortness of breath of two weeks duration. The patient had a four year history of progressively increasing alkaline phosphatase of etiology for four years. There was no history of significant alcohol abuse or risk factors for viral hepatitis. On examination, his sclerae were icteric and there were spider naevi on the back. There was decreased air entry and dullness to percussion in the right lower lung field. Abdominal examination did not show organomegaly or free fluid. Labs showed direct bilirubinemia with a total bilirubin of 11.7, alkaline phosphatase 1261, AST 122, ALT 254, albumin 2.4 with normal platelet count and coagulation profile. Chest X-ray showed moderate pleural effusion (Figure 1). ERCP revealed stricturing in the proximal and distal intrahepatic ducts with a beaded appearance. Common bile duct brushings were negative for malignancy. Liver biopsy showed findings suggestive of primary sclerosing cholangitis, prolonged obstructive biliary tract disease and moderate portal-periportal fibrosis. CT thorax and echocardiogram were negative for any mediastinal, pulmonary, pleural or cardiac pathologies that could cause pleural effusion. Thoracentesis revealed a transudative fluid with a serum-pleural fluid albumin gradient greater than 1.2, suggesting a diagnosis of hepatic hydrothorax. CT abdomen obtained at the time of admission did not show any ascites. The interesting aspect of this presentation was the absence of clinical or radiographic evidence of ascites.

The pathophysiology of hepatic hydrothorax involves the leakage of ascitic fluid from the peritoneal cavity into the pleural space through the embryologic defects, which are more prevalent in the right hemidiaphragm [1]. These defects arise from the rupture of pleuroperitoneal blebs as a result of raised intra-abdominal pressure from coughing, straining or ascites. The negative intrathoracic pressure aids the unidirectional flow of ascitic fluid to the pleural space through these defects. Diagnosis involves exclusion of cardiopulmonary disease, presence of transudative pleural fluid and demonstration of diaphragmatic defects when possible. The aim of treatment should be to relieve symptoms and prevent pulmonary complications by way of sodium restriction, diuretics, and therapeutic thoracentesis. Placement of a chest tube should be avoided as it leads to uncontrollable fluid loss and increased mortality. In resistant cases, pleurodesis with continuous positive airway pressure, videothoracoscopic repair of the defects [2], and TIPS may be considered until a liver transplantation is performed, which is the definitive treatment.

## Abbreviations

ALT: alanine transaminase
AST: aspartate aminotransferase
CT: computed tomography
ERCP: endoscopic retrograde cholangiopancreatography
TIPS: transjugular intrahepatic portosystemic shunt

## References

[bib-001] Lazaridis KN, Frank JW, Krowka MJ, Kamath PS (1999). Hepatic hydrothorax: pathogenesis, diagnosis, and management. Am J Med.

[bib-002] Huang PM, Chang YL, Yang CY, Lee YC (2005). The morphology of diaphragmatic defects in hepatic hydrothorax: thoracoscopic finding. J Thorac Cardiovasc Surg.

